# The effects of the COVID-19 pandemic on the well-being of children with autism spectrum disorder: Parents’ perspectives

**DOI:** 10.3389/fpsyt.2022.913902

**Published:** 2022-07-25

**Authors:** Aida Amirova, Anna CohenMiller, Anara Sandygulova

**Affiliations:** ^1^Graduate School of Education, Nazarbayev University, Nur-Sultan, Kazakhstan; ^2^Department of Robotics and Mechatronics, School of Engineering and Digital Sciences, Nazarbayev University, Nur-Sultan, Kazakhstan

**Keywords:** autism (ASD), children, parents, pandemic (COVID-19), online, inclusion

## Abstract

The COVID-19-related lockdown interrupted children’s learning progress and discontinued social learning and regular activities that children with autism spectrum disorder (ASD) rely on socially and physically. Negative consequences for children with ASD were reported far and wide. To investigate this problem in Kazakhstan, we conducted a mixed-methods study that drew on data from an online survey with 97 parents and semi-structured interviews with 14 parents. While parent-report quantitative results suggest that children were likely to experience negative impacts of the pandemic due to disrupted educational and therapeutic services, qualitative findings confirm that they have experienced an elevated mental health and behavioral challenges during the lockdown. Remote educational and therapeutic services were not helpful as families coped with pandemic-caused problems on their own. We highlight that continued support and care during and after a crisis is vital not only for children with ASD but also for the families under-resourced mentally and socially.

## Introduction

For nearly 2 years now, the COVID-19 pandemic has affected every aspect of life and continues to redefine the ways people socialize far and wide. Even before the current pandemic hit, children with special educational needs and disorders (SENDs) were among the underserved population with fewer opportunities to thrive in day-to-day life. Due to the pandemic-created social restrictions, children with SENDs and their families may experience unstable mental health and abrupt changes in mood and behaviors, as found widely in the United Kingdom (1, 2). In particular, research worldwide shows that persons with autism were severely affected by the current pandemic [Colizzi et al. (3) in Italy; Srinivasan et al. (4) in the United States; Kawaoka et al. (5) in Japan]. For example, a significant amount of studies have reported on the negative repercussions such as interrupted daily routines and increased psychiatric problems (e.g., irritability and sleep disorders) caused by the COVID-19 pandemic on the well-being of individuals with autism spectrum disorder (ASD) and their families [see Amaral and de Vries (6), Levante et al. (7), Vasa et al. (8)].

Children with ASD were particularly vulnerable during the pandemic because of their social, communicative, and executive functioning differences and co-existing conditions, such as physical and intellectual impairments (3, 9–11). For those with ASD, a surge in severe mental health conditions can be triggered by ineffective time management, unstructured activities, and disrupted daily routines in the home environment (7, 12). For instance, after two months of the COVID-19-induced lockdown, about 6 in 10 children with ASD reportedly worsened psychiatric conditions in the United States (8). These findings reveal that children’s behavioral problems may appear in more severe and intense ways during the global lockdown, and show significant negative consequences for parents as well. For instance, Morris et al. (12) explained this through the emotion contagion mechanism, according to which parents’ negative emotions, in particular, translate to their children during stressful situations like a pandemic. It is unknown what kind of influence COVID-19 and the lockdowns had and continue to have on children with ASD to address the educational and therapeutic challenges that may emerge in a post-pandemic future. As a result, research studies attempting to understand the impact of COVID-19 on the social well-being of the children, whether positive or negative, are called for (6, 12). Indeed, the effects of the pandemic may vary from country to country due to different autism contexts and the spread of COVID-19. Therefore, we first present how autism is understood and treated in Kazakhstan and later provide a COVID-19 timeline.

### The context of autism in Kazakhstan

Kazakhstan, a post-Soviet country, is undergoing a massive shift in creating an inclusive society, once unbeknownst to the majority as a concept. In Kazakhstan, ASD is poorly understood as a developmental impairment (13). The legal framework does not include the term “ASD” with respect to disorders in official documents, partly because they group children with special needs or mental health impairments under the same group of disorders (14). This generalization of developmental and mental impairments may delay diagnosis and consequently treatment. Children with physical and mental barriers are being treated with a deficit mindset. A recent Kazakhstani study shows that individuals with autism continue to face stigma and discrimination from other people (15).

According to official statistics (available since 2010) in Kazakhstan, one or two in 100,000 children were diagnosed with ASD from 2010 to 2012 (16). However, there are contrasting data on the number of children diagnosed with ASD and receiving the treatment. There is no reliable and publicly available data on how many children and adults live with this diagnosis in the country. Most of the information we report in this section comes from media sources. According to the latest data from the Ministry of Health, the number of children with autism has increased more than 20 times in the last 13 years.^1^ They also reported that the screening tests such as ADOS – 2 и ADI-R had been introduced only recently. Children with ASD receive treatment through a special support scheme Psychological-Medical-Pedagogical Commissions (PMPCs), which are predominantly under-resourced, and practitioners are often oblivious of the latest developments in autism diagnosis and treatment (17). Furthermore, despite some improvements to integrate students with special needs in the traditional classrooms, most students still go to the so-called correctional or remediation classes or study in separate “inclusive” classes within mainstream schools (18). Pedagogical institutions have introduced new modules on inclusive education in the curriculum of pre-service teachers. According to the Ministry of Education, the number of teaching assistants and staff with special qualifications is increasing.^2^ Inconsistent autism diagnosis, the lack of legal framework, and support in terms of social care and education provision prevent people on the autism spectrum from living in a more inclusive and equitable society. Of course, the challenges Kazakhstan faces in implementing inclusive education are also found in other contexts (19).

Some promising reforms are to be implemented. In particular, we have noticed several grassroots-level campaigns led by parents of children with ASD. We believe that they are accomplishing truly significant achievements bringing together other parents and thus expanding their network and visibility. For example, a mom of an autistic child started the National Association ‘‘Autism Kazakhstan’’^3^ and advocates for the rights of the children living with developmental disorders, including autism. Another great project is ‘‘Autism Pobedim’’^4^ which organizes educational, therapeutic, and recreational activities for children with ASD. In addition, there are regional autism centers such as ‘‘Asyl Miras’’ funded by Bulat Utemuratov Fund.^5^ Here, children with ASD until the age of 15 can receive free educational and therapeutic support from professionals. Despite these significant improvements, there remains much work to be done. More research is needed to support the activities of these organizations and further identify effective mechanisms, best practices, and sustainable solutions to scale up.

A roadmap for the provision of care for children with autism was introduced for multiple purposes, including to collect data on the child population diagnosed with autism and other mental disorders, to increase the provision of specialists, build a national Autism Center, and analyze and improve state laws and documents related to the status of children living with neurodevelopmental disorders (20). However, the current status is unknown. These activities are highly needed to recognize the needs of families raising a child with ASD. As such, these new steps reinforce the importance of continuing autism research to provide better outcomes that guide traditional and alternative ways of support for the healthcare system and clinical professionals to meet effective decisions.

### The COVID-19 pandemic in Kazakhstan

**FIGURE 1:**
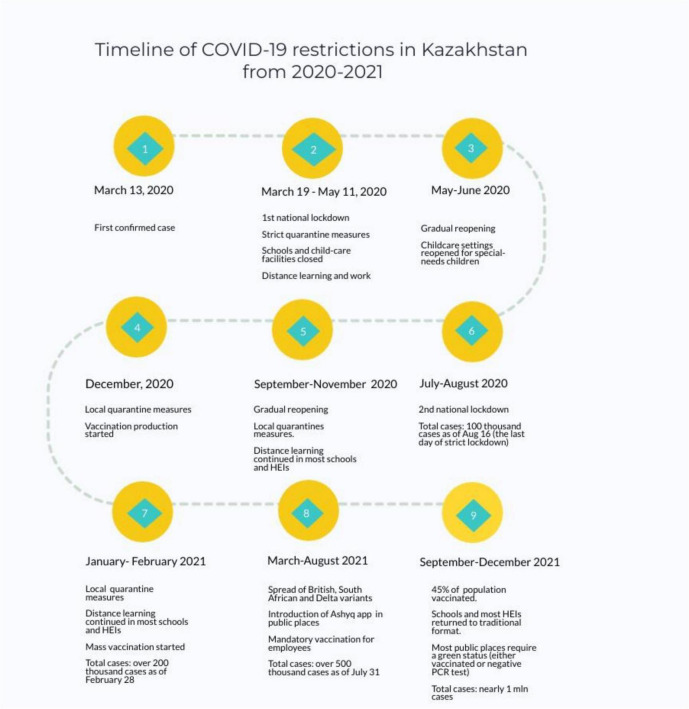
Timeline of COVID-19 restrictions in Kazakhstan from 2020 to 2021.

The World Health Organization (WHO) declared COVID-19 a pandemic on 11 March 2020; by that time over a hundred thousand cases were registered worldwide (21). The pandemic hit Kazakhstan at about the same time world countries announced the state of emergency (see Figure 1). A national “lockdown” started on 19 March 2020, in two major cities, with a later announcement for lockdown in other regions. All educational institutions, including special-care centers were required to stop all services on the same day the lockdown began. School-aged children had early spring break 2 weeks before the abrupt change to learning remotely in April 2020. Similar to other countries, the transition to remote education was *ad hoc* and often unsuccessful due to extensive gaps in digital access and skills in the country.

In May 2020, children with special needs were allowed to visit daycare centers and treatment facilities upon parents’ consent to follow the restrictions set by the facilities. In fact, Kazakhstan experienced the first wave between July and August 2020, when other parts of the world as many countries in Europe started to gradually reopen their economies after the first shocking COVID-19 outbreak in Spring 2020. Although the country eased quarantine measures from Fall 2020, there have been significant fluctuations in daily cases, while quarantine measures have been still in place for most public activities. In Fall 2020, most schools and universities did not reopen, leaving online learning the only choice for many. While the mass vaccination started early in February 2021, the number of vaccinated people remained low until summer 2021. Vaccination has been accelerated due to mandatory workplace requirements and the implementation of a tracing app (Ashyq) to enter public places. All schools returned to traditional face-to-face learning in September 2021, yet some schools were closed when the school-wide COVID-19 situation was out of control. According to Ministry of Health data, a total confirmed cases of COVID-19 stand at about one million people. Of them, a total of 7,291 children were registered with a confirmed diagnosis of COVID-19 in 2020. Since the beginning of 2021, about 45,000 cases of coronavirus infection among children have been registered in Kazakhstan, a sevenfold increase compared to the whole period of 2020.

### The current study

In the past decade, Kazakhstan has increased its awareness of autism and initiated special support mechanisms to support children with ASD and their families. However, the 2 years of the pandemic thwarted that progress and disproportionately affected countries and their social care systems. More active commitment to health care and education is needed to learn though this experience and prevent the same challenges from happening in future. Considering all of the above, we seek to explore how the COVID-19 pandemic influenced the well-being of children with ASD, as reported by their parents. Specifically, we seek to understand how parents of children with autism perceive the impact of COVID-19 on child behaviors, treatment, and daily life in general. The study reveals some profound insights into the importance of continued care for children on the autism spectrum and the need for support mechanisms to serve their needs in times of crisis and beyond. This study contributes to the growing body of autism research conducted worldwide during the pandemic to enable system-wide and evidence-informed decisions and support mechanisms for both children and their parents during emergencies.

## Materials and methods

This mixed-methods study was conducted using an online survey and qualitative interviews. Ethics approval for this study was obtained by the Nazarbayev University Institutional Research Ethics Committee. Informed consent forms were collected before taking the survey and participating in interviews. All participants were provided specific information about the research purpose, procedures, benefits and risks, privacy considerations, potential contribution, and contact details of the principal investigator.

### Survey

The survey was developed on Qualtrics. It was available from August to September 2021. The survey and interview questions were piloted with one parent *via* Zoom and adapted based upon feedback. To ensure privacy and confidentiality, settings did not collect personal identifiers such as names, contacts details, and IP addresses. That is, the survey link was anonymized. There were no forced-choice questions, and instead, participants were allowed to leave the survey at any stage. There were 26 questions in total in the survey (see Supplementary Material) which included data demographics about the child and parent and the effect of the COVID-19 pandemic on child wellbeing. Here, the human/subjective well-being is defined as a multidimensional construct combining psychological, physical, and social dimensions (22, 23) in which self-report measures and ratings are indispensable (24). Our understanding draws on subjective parent-report data that indicate how child conditions such as mental health and social behaviors are affected by a novel pandemic. We self-developed survey questions to assess “well-being” through 5-point Likert scales. It included agreement scales, perceived mood rate, satisfaction, therapy quality, importance of educational, and therapeutic services. The surveys were bilingual so that parents could choose the preferred language, Kazakh or Russian.

### Interviews

The interviews were conducted concurrently with the survey. Participants who wished to participate in the follow-up interview provided their email or telephone number at the end of the survey for us to contact them. The parents could choose the preferred language, Kazakh or Russian. Interviews consisted of 16 open-ended questions (see Supplementary Material). They lasted between 17 to 60 min, with an average of 30 min. Prior to the interviews, all parents filled out the consent forms. All parents requested to be interviewed *via* WhatsApp audio call rather than Zoom, mostly due to technical constraints. The researcher also asked for verbal assent before recording the conversation. Names, locations, and other identifiers were masked by applying pseudonyms. We used pseudonyms when presenting the interview results, and any resemblance to actual names is a mere coincidence. The interviews were conducted either in the Kazakh or the Russian language.

### Participants

Participants were recruited using convenience and snowball sampling. To recruit participants, we first contacted parents with whom we worked earlier on studies of robot-assisted therapy for children with ASD (25, 26). As we needed to recruit more participants for the survey, we reached out to potential gatekeepers, such as the parental community, autism centers, and non-profit organizations, *via* email and Facebook. We contacted them directly using the details on their official sites. In particular, the local network of autism centers helped us send out the anonymous survey link to parents’ groups and then recruitment flyers (see Supplementary Material). A total of 109 parents of children with ASD in Kazakhstan participated in the survey. Considering that 12 participants did not respond to any question, a total of 97 respondents were included in the final analysis. The mean age of the parents is 34.7 (SD = 6.8). Of 97 participants, 14 parents took part in the semi-structured interviews (see Table 1).

**TABLE 1:** Demographics of parents and children.

	*N*	(%)
**Parents**		
Gender		
Female	95	98
Male	2	2
Age range		
Young (<30)	22	22.7
Middle-age (30–39)	54	55.7
Older (≥40)	21	21.6
Place of living		
Urban	84	86.6
Rural	13	13.4
Parents’ education		
School	15	15.8
Vocational	13	13.7
University	64	70.5
Employment status		
Employed	58	60.4
Unemployed	38	39.6
Home language		
Kazakh	24	25.5
Russian	39	41.5
Two languages	31	33
**Children**		
Gender		
Female	27	29.3
Male	65	70.7
Age range		
≤5	36	40
Ages 6–8	36	40
≥9	18	20
Age range at diagnosis		
≤2	13	20.6
Ages 3–4	33	52.4
≥5	17	27
Schooling		
No formal schooling	25	28.1
Mainstream schooling	20	22.5
Special centers	44	49.4
Therapy status		
Therapy	77	86.5
No therapy	12	13.5
Siblings		
Siblings	77	79.4
No siblings	20	20.6

#### Quantitative analysis

Quantitative data obtained from the online surveys were analyzed with SPSS. A series of Kolmogorov–Smirnov tests were conducted to check if the data were normally distributed. We ran Mann–Whitney U and Kruskal–Wallis tests for non-normally distributed data. Predictor variables included: urban–rural, parents (education background and employment status), family (language background and number of children), and children (gender, current age group, age diagnosed with ASD, schooling status, and access to therapy before and after pandemic).

#### Qualitative analysis

Qualitative data collected from interviews were analyzed using thematic analysis (27, 28). We used inductive and deductive coding strategies (29, 30). Themes and codes were checked for accurate interpretation of results by consulting interview excerpts. The interview questions can be found in Supplementary Material. The interview findings were transcribed and then coded by hand using thematic analysis. One researcher analyzed over 500 min of audio recordings. The emerging themes are related to the impact of quarantine measures on children with ASD and their families, as shown in Table 2.

**TABLE 2:** Emerging themes.

Themes	Quarantine measures and restrictions	Effects for children and their families	Remote therapy/learning
**Sub-themes**	1. Access to daily services 2. Restrictions to therapy/learning 3. Child understanding of quarantine measures 4. Parental fear and challenges 5. Home environment	1. Behavioral 2. Psychological 3. Physical 4. Family bonding	1. Challenges with instruction 2. Child inattention 3. Remote learning 4. Support mechanisms

## Results

In this section, we provide first the results from an online survey and then move on to discuss interview findings.

### Survey results

#### Perceived impact of the COVID-19 pandemic

**FIGURE 2:**
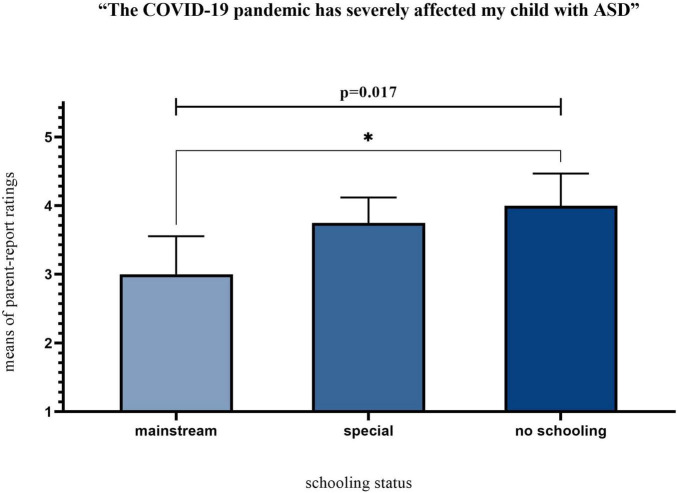
Perceived impact of the pandemic grouped by schooling status. *Means statistically significant pair-wise comparisons.

Parents reported an agreement score of 3.65 (SD = 1.21) (out of 5 for “Strongly Agree”) for the question of how severely the COVID-19 affected their children with ASD. To explore what socio-demographic characteristics of the surveyed families differentiated their responses, we conducted a series of Mann–Whitney U and Kruskal–Wallis tests. The majority of independent variables did not reveal any statistically significant results. However, we found a statistically significant difference in the perceived impact of the pandemic between respondents with children of varying schooling status: χ^2^(2) = 8.199, *p* = 0.017. Parents of children without formal schooling reported a significantly stronger impact of the pandemic (4.00 ± 1.09) compared to those parents of children that attended mainstream schools (3.00 ± 1.15), as presented in Figure 2.

#### Child and parent mood during the pandemic

**FIGURE 3:**
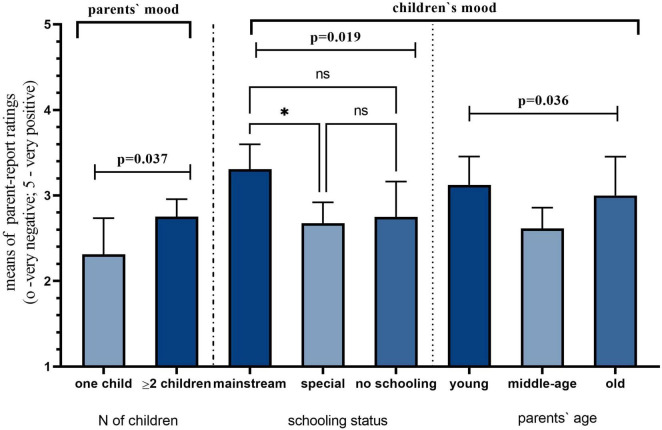
Child and parent mood during the pandemic. Asterisk indicates a 0.05 level of significance.

The mean of parents’ mood was 2.67 (SD = 0.851) out of 5, with 0 being very negative and 5 being very positive. Interestingly, there was a significant difference in parents’ mood between the reported number of children: *U* = 721.000, *p* = 0.037. Parents with two or more children had significantly higher levels of positive mood (2.75 ± 0.847) than those with only one child (2.31 ± 0.793). Parents reported their child’s mood to be 2.81 (SD = 0.753) out of 5 with 0 being very negative and 5 being very positive. It was significantly predicted by parents’ age groups [χ^2^(2) = 6.631, *p* = 0.036] and children’s schooling status [χ^2^(2) = 7.957, *p* = 0.019]. Children of young parents aged less than 30 years old had higher mood ratings (3.13 ± 0.619) than children of older (3 ± 0.784), and middle-aged (2.62 ± 0.747) parents. Another interesting finding is that children attending mainstream schools were reported to have higher levels of positive mood rate (3.31 ± 0.480) than their peers in special centers (2.68 ± 0.764) and those without any formal education (2.75 ± 0.775). Pairwise comparisons showed significant differences between children at special centers and mainstream schools, as shown in Figure 3.

#### Frequency and satisfaction with autism spectrum disorder therapy before and during the pandemic

**FIGURE 4:**
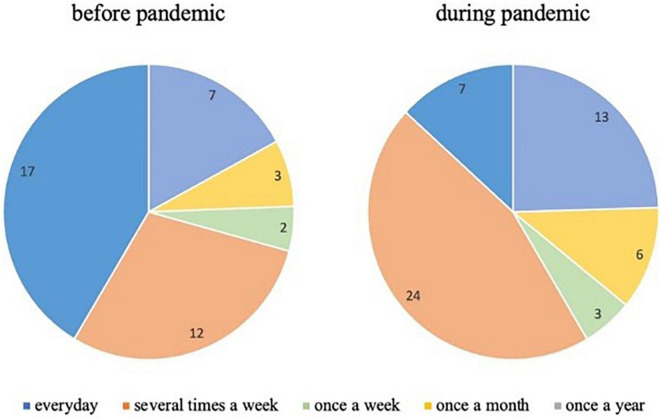
Therapy frequency in pre-pandemic and pandemic periods.

We found no significant differences in the frequency of ASD therapy before and during the pandemic. The small sample size might explain this. However, there is a marginal difference between receiving the treatment in both periods. The frequency of everyday treatment dropped after the pandemic from 17 to 7 as conducted at the treatment center. There has been a double increase in the category “several times,” from 12 before March to 24 after March 2020 (see Figure 4). The number of treatment sessions slightly increased in other categories, which shows a greater need for therapeutic services during the pandemic. However, the paired samples *t*-test showed that the parents’ satisfaction with ASD treatment decreased significantly during the pandemic (3.12, SD = 1.142) compared to the pre-pandemic time (3.42, SD = 1.136): *t*(58) = 2,125, *p* < 0.050.

#### Quality of online autism spectrum disorder therapy during the pandemic

**FIGURE 5:**
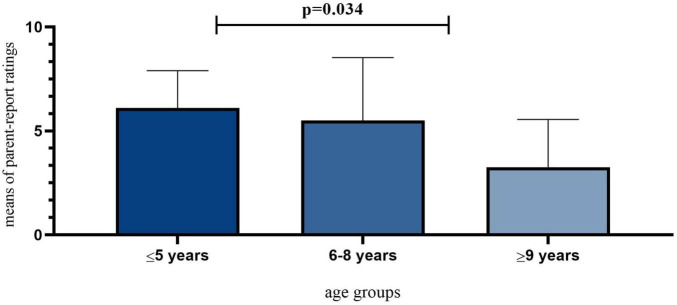
Quality of online ASD therapy during the pandemic.

Overall, the satisfaction rate with the quality of ASD therapy was low and stood at 4.87 (SD = 2.876) out of 10. The results show a highly significant difference in the reported quality of online ASD therapy depending on children’s age groups: χ^2^(2) = 7.038, *p* = 0.034. Parents of children aged five or younger rated online services more favorably than those having children in older age groups (see Figure 5).

#### Support mechanisms during the pandemic

**FIGURE 6:**
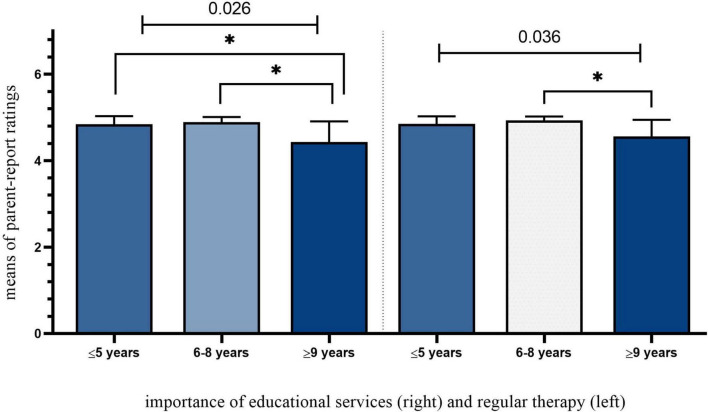
The importance of support mechanisms. *Significant pair-wise comparisons.

We also tested how parents rated the importance of support mechanisms in terms of access to therapeutic and educational services (see Figure 6). We found clear trends in the ratings of the importance of educational services [χ^2^(2) = 7.311, *p* = 0.026] and regular therapy [χ^2^(2) = 6.626, *p* = 0.036] between children’s age groups. Parents of children aged nine and older rated that those services were significantly less important than parents of younger age groups. Compared to older age groups, parents of children aged 6–8 rated regular therapy and educational services significantly more important.

### Interview findings

#### Theme 1: Coping with quarantine measures and restrictions

##### Pandemic-caused restrictions in daily life

Self-isolation was exercised widely, requiring people to stay at homes to contain the spread of COVID-19. It has been discouraging for many people, but our findings show that the stay-at-home measure heavily influenced children with ASD. They are particularly vulnerable to the quarantine measures that restrict access to educational, recreational, and therapeutic services because their well-being depends on regular social and health support. A recurrent concern for all families was restrictions applied to free movements when the national lockdown was announced. Parents reported that their children were frustrated while staying indoors. These restrictions were lifted after some time in quarantine, and children with special needs like autism could go outside, as illustrated in this quote:

When the pandemic began, the only difficulty was that we could not go out after 6 p.m. Once when we were walking in the playground, a police officer, who was doing a quarantine compliance check, told us to go back home and not walk around. We were following all the quarantine measures and wearing a mask though. It was only then [after lockdown] that children with special needs were allowed to go outside (Alena).

Some emergency services were limited as cities had to set up special quarantine checkpoints, which prevented any transport from crossing designated places. For instance, Aidana shared her experience of calling an ambulance from the city, which was returned to the city: “Once my son hurt his leg when he rode the scooter inaccurately and fell down. We called an ambulance. *At the checkpoint, they [police officers] did not allow the ambulance to pass* because it came from the city.” (Aidana)

##### Parental concerns over children’s health

A common view among the parents was that they endured higher fear levels concerning children’s health during the quarantine. Such fear likely stems from the medical observation, according to which children with ASD are commonly diagnosed with co-occurring health problems (e.g., ADHD). Such stress and fear are illustrated in the comment below: “My child has primary immunodeficiency disease (PIDDs) and other diseases like VRI intolerance. When quarantine announcements began to appear, a week before the quarantine, we already canceled lessons [with the specialist]. I feared for her health” (Galiya). Besides health issues, parents had to restrict outdoor activities for other reasons. As one parent stated, her family moved into a new district during the quarantine: “Last year [during the quarantine], we movedy to a new district. It was a new place. We didn’t know our neighbors. Thus I didn’t let my child out. We didn’t go outside during the quarantine.” (Aliya)

##### Home environment

We found differences in quarantine experiences of families that reside either in private homes or apartment buildings. The impact of variations in home environments during the quarantine was tangible. Some parents lived in single-room apartments that further affected their life in quarantine and their children’s well-being. The following quotes describe these experiences vividly of their children with ASD not having sufficient space for physical and emotional needs: “As we lived in a single-room apartment, we were restless and couldn’t find peace.” (Zhansaya); “During the quarantine, we were stuck in a single-room apartment with an autistic child and a toddler. It was an unpleasant experience; even a typically developing child got bored.” (Mira)

#### Theme 2: Effects for children and their families

##### Negative effects of the quarantine on children with autism spectrum disorder

Hyperactivity was a regular condition as children showed atypical behaviors that surprised and concerned their families: “she [tried] climbing ceilings” (Dariya), “he ran, jumped, turned around, did not comply” (Zhansaya), “he ruined everything he touched” (Aisulu). Predominantly, there was a combination of such behaviors. Sabina reported the following:

After about 1.5 months at home […] My eldest son with autism cried every day. I did not understand what was wrong, so I asked him what had happened and what had hurt. There was aggression in addition to crying. He could not calm down.

While for most children the reason for their negative behavior was the lack of time outdoors, a few children had to spend time with people who were unaware of approaches to helping children with ASD:

He spent summer in the village last year [during the quarantine]. He forgot the skills he learned. In September, when he returned, he had aggression and tantrums because he didn’t want to study in the [autism] center. When he was in the village, no one demanded anything from him because his grandmother didn’t know how to deal with him. (Mira)

A substantial number of parents reported increased screen time on gadgets and TV. This subsequently resulted in the lack of attention to the surrounding environment. Most children seemed to get stuck in such events leading to such extreme instances of not eating:

Since we didn’t go outside last year, we gave her the phone we took from her earlier [in the year]. Then she was stuck on the phone and didn’t look around. She didn’t care whether we came by or not. She was enough for herself. She didn’t even eat food. (Aliya)

Although the majority of mothers were housewives with a spouse/family to financially support the household, some were the primary breadwinner, working and supporting their children on their own. Those parents were desperate to rely on gadgets to keep their children engaged while working from home during the quarantine. This is clearly discussed in the following comment:

I worked online, and the kids were on their own. To ensure that they didn’t scream and fight, I gave the older one [with ASD] the phone, and the [TD] younger one took care of himself. The child [with ASD] got addicted to the gadget because of it. Certainly, it shouldn’t be that way. But when you have kids to feed, you have to work (Mira)

##### Positive effects of the quarantine on children with autism spectrum disorder and their families

Despite the adverse consequences of the pandemic, a few children improved their behaviors and learned new skills while at home. However, such progress should not be only associated with the pandemic but also with their development. The following quotes refer to the improvements, such as fewer extreme behaviors, more compliance, and new learning: “I mirrored his actions when he screamed and stomped his feet at home. I asked older children to do the same. He somehow started to understand that those actions were not good. Now he does them less often.” (Aisara); “He started to comply with my instructions a bit. He began to use his forefinger. He couldn’t show it before. He also started to pronounce some words like ‘Dad,’ ‘Mom’ and ‘Give.”’ (Zhansaya); “Primarily, we practiced fine and gross motor skills. He can hold the pen; until then, he held it like a shovel. Now he holds it with three fingers as it should be.” (Aliya)

In our study, almost all families had two or more children. The participating parents reported that life in quarantine was helpful to reconnect with family members and understand each other, contributing to their children’s well-being. In normal times, fathers or siblings seem not to be as close in physical proximity and emotionally as mothers do with their children with ASD. However, during the pandemic, with parents working from home, some parents commented on a positive change in family values: “Most of all, I liked that my family reunited as if we understood each other better. Family values became tangible… We were constantly engaged. For instance, we performed physical exercises and played games with the child [with ASD] just to keep him interested. *My husband and everyone [siblings] contributed, not just me alone.*” (Aidana); “The positive thing is that her dad got to spend more time with her because he was in quarantine, too” (Galiya). Dilyara reported the importance of parental involvement in delivering remote learning:

[During remote learning], we became our child’s teacher and taught him, for example, all kinds of football activities. We video-recorded him at the same time. Two parents must be involved. One of them explains everything to the child, where to score the ball, how to do the exercises, while the other has to record the process. (Dilyara)

Almost all parents explained efforts to develop their children’s social and life skills regularly. They reported that their children became more independent and skillful to fulfill their own self-care needs. For instance, with fewer cars and people on the road, Dariya could teach his son street safety. “Last year during the quarantine, I taught him how to behave in the streets, like ‘’Stop,’ ‘Look out,’ ‘Wait, there’s a car.’ I taught a lot during the quarantine because there were a few people outside.” (Dariya)

In other cases, children mastered self-care skills themselves, as reported by Alena:

Positive changes related to self-care and hygiene like washing hands. We attended remedial kindergarten for two years, but it may happen that he hadn’t been taught. He probably was still a little child back then. But during this time [quarantine], when we stayed at home, my child learned how to take care of himself and be independent, so he could take care of himself and dress himself. (Alena)

#### Theme 3: Experiences with therapy and learning during the pandemic

##### Remote therapy challenges

Almost all parents received asynchronous support from teachers and specialists to whom they sent out the tasks *via* social networks like WhatsApp. Technical constraints made it difficult for teachers to observe children’s behaviors online. Therefore, this format is not sustainable for children with ASD, says one parent.

We had a WhatsApp video call for 30–40 min or so. The only thing was that I did everything without a therapist alongside. The teacher did not fully observe the child because I put the phone on from a different angle. The observation gets worse because of the video call (Galiya).

Some parents thought online therapy was better than nothing, supporting their child on their own relying on therapeutic support online. But the therapy time decreased a bit, likely due to restrictions set on platforms like Zoom, limiting free accounts to 45 min.

I was happy to have online therapy because we didn’t waste time. Even though my child didn’t participate directly, *I learned and taught my child myself. But offline [treatment] is better.* Due to the pandemic, therapy time decreased to 45 min; earlier, it was one hour. (Aliya)

For some children with ASD, their sensing abilities are a significant source of their learning processes, which could disadvantage them during online therapy: “I don’t think it’s [online therapy] functional because autistic children are susceptible. They need visual and tactile contact. My child likes touching everything.” (Zhazira)

##### Remote learning challenges

It would not be an overstatement to say that online learning was of great tension for children with communication and attention barriers distinct to autism. All parents unanimously were against adopting online learning as an alternative to traditional learning. One parent described her child’s experience in an online dance club as a failing experience:

It’s us – a dad and a mom – who do tasks while online. It can’t be a lesson. He didn’t understand anything and had no interest at all. Therefore he quit the dance club. In-home and online dancing was challenging. He could perform physical activities but not dance movements. (Dariya)

With remote learning, parents had to fill in for the newfound roles of teachers for their children. What works for some children may not work for those children with special needs, and many parents were in a trying situation during the pandemic. Two parents confessed that they wanted to remain mothers, not teachers. For instance, a child showed negative attitudes toward his mother who had to act as a teacher:

I want to remain a mom to my child… During the quarantine, I demanded him practice motor skills. I made him write, draw, and imitate, which he needed to do based on his age range. I tried to do those activities in the Centre by myself. But it was not good for us. It backfired because he started to develop negative feelings towards me. For such children, it is better to do activities with a stranger, not their mom. (Lara)

Child inattention seemed to be a major reason for the unsuccessful transition to online learning. For children with ASD, it is hard to keep undivided attention to activities. In most cases, children tend to perceive online learning unseriously because they are usually exposed to technologies such as a mobile phone as a source of entertainment to watch videos or play games: “Online learning is not applicable because the child sees the phone, and he loses engagement. He wants to watch YouTube. Otherwise, he screams hysterically” (Mira). One parent even perceived it causing an opposite effect:

We certainly had a few online classes. But it did not work. He was freaking out that he was not allowed to watch what he wanted and instead was forced to watch the classes online. It was stressful for him and for us. We gave up on online classes because they had no effect. It was even the opposite. It was detrimental. (Lara)

##### Key findings

• Pandemic-caused restrictions disrupted access to essential and non-essential services, particularly the access to therapeutic and medical facilities that are vital for children with ASD. In particular, the overall well-being of children with no schooling experience were likely to worsen compared to those in mainstream schools during extended lockdowns.
• Both parents and children had reportedly lower mood ratings during the pandemic. Parental concerns over a child’s health and safety were elevated because some children have co-occurring conditions such as immunodeficiency and anxiety, while the imposed restrictions in daily life triggered the atypical behaviors among the children.
• Although we collected data on the employment status of participants, it did not reveal any significant differences in the perceptions of employed and unemployed parents. Yet, the qualitative findings show that families from lower socioeconomic backgrounds had fewer opportunities to support their children while at home. In the study, most families raising a child with ASD have two or more children and usually reported to struggle to have ends meet.
• The time allocated for most free treatment services was shortened. Online therapies were also short partly due to time restrictions in platforms like Zoom. The number of daily treatment sessions marginally dropped threefold when the pandemic hit.
• Parents reported distance learning experiences discouraging and not helpful both for their children and themselves. Although parents attempted to replace teachers and therapists at home, they had no training to serve their children’s needs in the best possible ways. Parents’ satisfaction with ASD treatment decreased significantly when we compared how parents rated their satisfaction in the pre-pandemic and the pandemic period.
• Both educational and therapeutic services were found to be unanimously important during the pandemic. This finding clearly explains how salient is the access to key support mechanisms for children with ASD; as Baweja et al. (9) noted, “it takes a village” to raise a child with ASD. The quality of online ASD therapy was rated notably low. It might be affected by how unprecedented the shift from traditional to online treatment was.

## Discussion

The study explored how children with ASD experienced the COVID-19 pandemic from their parents’ perspectives. We particularly investigated whether children with ASD are vulnerable to pandemic-imposed social restrictions that were found to bring overwhelming challenges to regular treatment and learning across countries. Most parents reported that the restrictions related to therapeutic and educational services were particularly prominent. We found quantitative evidence as represented in significant differences in the parent-report severe effect of the pandemic on children who did not have formal schooling. Interview findings confirm that the lockdown measures have resulted in disrupted ASD treatment, which is critical to achieving positive benefits in the long term (31–33).

The degree to which children with ASD in Kazakhstan experienced adverse effects of the pandemic is not uniform. We could not identify if there were differences in the perceived effects of the pandemic between employed and unemployed parents, particularly due to small sample size. But qualitative findings show varying home environments may affect considerably. For instance, parents living in small apartments reported fewer opportunities to support their children in regular practices of new skills, while those in private houses had resources and physical spaces to arrange recreational activities outside. This may account for a lower socio-economic situation in the families. For instance, low-income families tend to report greater struggles when caring for their autistic child during the quarantine. This confirms prior research emphasizing intensified inequities in coping with stress and behavioral changes between economically weaker and better-off families raising a child with ASD (8, 34–35).

The results show that technology-based autism treatment modalities cannot replace in-person services. That being said, our data further shows that only certain groups may have relatively positive online experiences, for instance, young children under 5 years old. This finding is entirely unexpected, however, the learning content for young children with ASD might be more entertaining and easy to follow in daily life compared to older children who need more advanced and complex learning guidance. For instance, parents of older children rated the importance of educational and therapeutic services relatively higher than that of younger children. Some inherent limitations with telehealth-based intervention mainly include child inattention during sessions and technical issues like video setup. This problem might not be unique to these children as their neurotypical peers may lose interest easily. Internationally, when children with ASD could not attend schools, many parents reported having online lessons and activities *via* digital platforms such as Zoom and Skype (12). In our study context, parents could only access asynchronous learning and therapy services through WhatsApp. In this case, therapists or tutors send out activities that children need to perform (e.g., physical activities and daily skills) while their parents/siblings either assisted or recorded children’s activities. The quantitative results also show that the parent-report satisfaction rate with the therapy decreased during the pandemic.

Distance learning environments are less likely to meet the needs of children with ASD if they are not given adequate guidance and support in managing the new environment they have never experienced before. Since rehabilitation centers and schools were partially or fully closed, parents of children with ASD had to replace teachers to continue education from home. Our study suggests that parents of children with ASD should be supported more during crises. Some parents reported being exposed to elevated stress due to the quarantine and its discouraging impacts. This experience was overwhelming for most parents – who are largely – not prepared to delegate demanding parental and educational responsibilities, to name a few. Parents were conditioned to care for their children on their own while juggling multiple responsibilities for which they had not been trained. The unique struggles of parents of children with ASD are echoed in similar studies that report family tensions (36), lacking a resourceful state (37), and increased stressors during the pandemic (38). Parents often need extra support from other people, particularly professionals who can aid and counsel when raising a child with a developmental impairment. It is true that without the challenges of COVID-19, parents usually face many obstacles in obtaining continuous care.

We conclude that children with ASD and their parents endured mental and behavioral problems that seem to be predicted by disrupted therapeutic and educational opportunities. This is supported by the quantitative results reporting higher agreement with the perceived adverse effects of the pandemic on both children and parents. Almost all parents reported that remote educational and therapeutic opportunities did not satisfy their children’s needs due to the challenges associated with unique autistic traits, technical problems and parental stress. System-wide and evidence-informed support mechanisms for both children and their parents need to be implemented to support telehealth therapy modalities.

The main limitation of this work is that we could not deliver standardized tests evaluating medical conditions such as anxiety level and other behavioral changes during the pandemic. Yet, to our knowledge, this is the first study that explored a relatively large sample of parents of children with ASD in Kazakhstan to date. This study could be extended by conducting a follow-up study on post-COVID treatment options and what has been learned after the series of lockdown measures. The results from the current study can help identify therapeutic needs of the children in future interventions embracing remote technologies and in-home treatment options for parents and other caregivers. More research and development are called for to advance the current limitations of therapeutic and educational opportunities in Kazakhstan and elsewhere.

## Data availability statement

The original contributions presented in this study are included in the article/Supplementary Material, further inquiries can be directed to the corresponding author.

## Ethics statement

The studies involving human participants were reviewed and approved by the Nazarbayev University Institutional Research Ethics Committee. The patients/participants provided their written informed consent to participate in this study.

## Author contributions

AA: data collection, data visualization, and writing the first draft. AC: conceptualization, and writing—review and editing. AS: conceptualization, supervision, funding acquisition, data curation, and writing—review and editing. All authors contributed to the article and approved the submitted version.

## Footnotes

1
https://24.kz/ru/news/social/item/464976-v-kazakhstane-v-20-raz-vyroslo-chislo-detej-s-autizmom

2
https://www.gov.kz/memleket/entities/edu/press/news/details/252469?lang=ru

3
https://ainar.org

4
https://autismpobedim.kz

5
https://utemuratovfund.org
