# Supplementary material for: The effects of the COVID-19 pandemic on the well-being of children with autism spectrum disorder: Parents’ perspectives
Source: Front Psychiatry. 2022 Jul 25;13:913902. doi: 10.3389/fpsyt.2022.913902 (PMC9359431; doi:10.3389/fpsyt.2022.913902)
Supplement: Supplementary file 1 [file Data_Sheet_1.docx]

Supplementary Materials

Recruitment flyers for parents


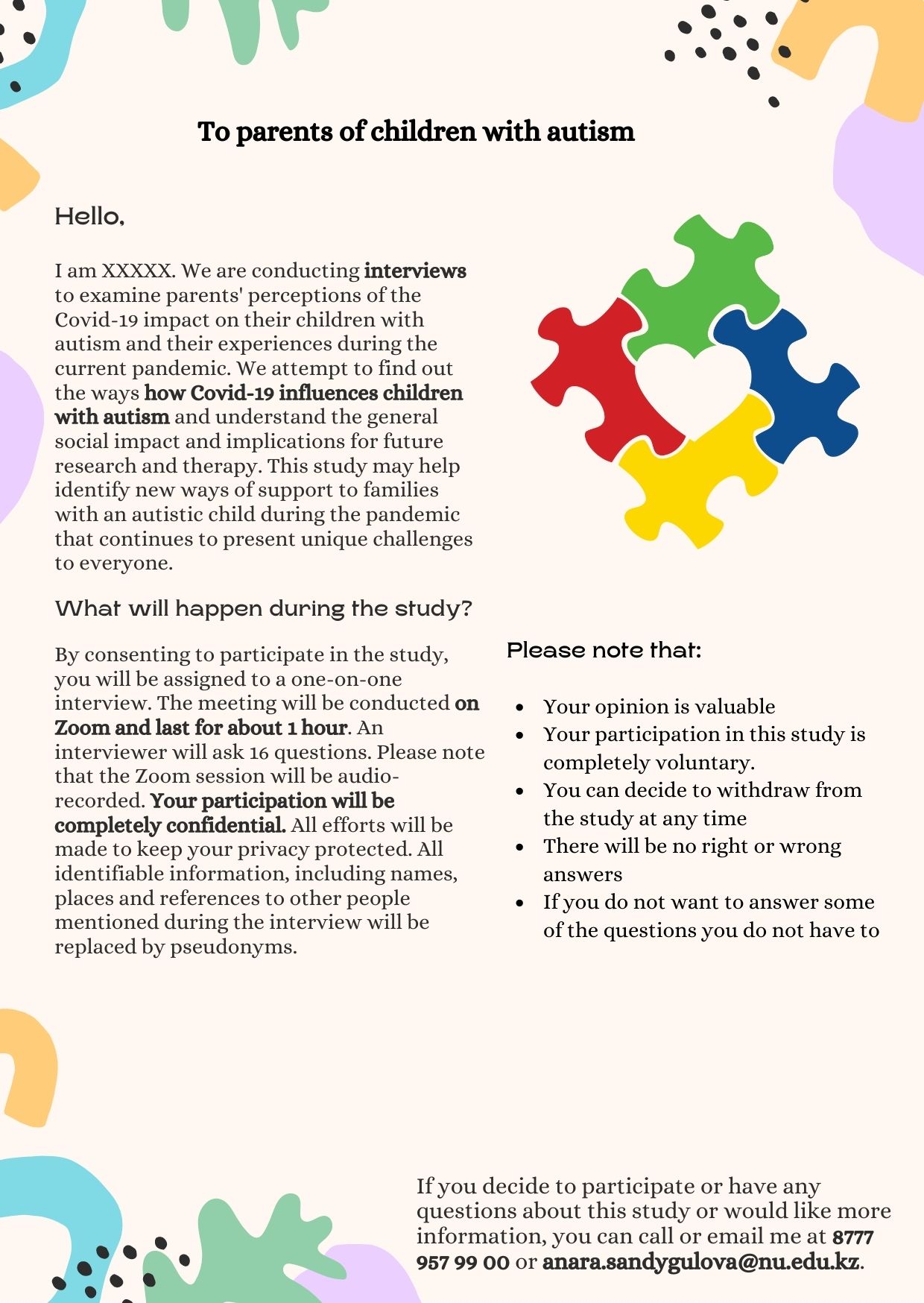


# Online survey questions

Q1  How old are you?

- 18-30
- 31-40
- 41-50
- 51-60
- 61 or older

Q2  What is your gender?

- Male
- Female
- Prefer not to say

Q3  In which area do you live?

- rural
- urban

Q4 What is the highest degree or level of education you have completed?

- High school
- Vocational
- Undergraduate
- Postgraduate
- Not applicable

Q5  What's your occupation status?

- full-time employed
- part-time employed
- Self-employed
- Not employed or not looking for a job
- Unemployed or looking for a job
- Retired

Q6 How many children are there in your family?

- one
- two
- three or more

Q7 What's your family's home language?

- Kazakh
- Russian
- English
- Other (please indicate)  ________________________________________________

Q8 How old is your child with ASD? Please indicate age in years and months (e.g. 4 years and 6 months) diagnosis age

________________________________________________________________

Q9 What is your child's gender?

- Male    gender
- Female
- Prefer not to say

Q10  At what age did your child get diagnosed with autism? (e.g. 4 years and 6 months)

________________________________________________________________

Q11  What kind of autism therapy does your child usually receive?

Therapy/ no therapy

- Speech and language therapy
- Psychological therapy
- Physiotherapy
- Music therapy
- Social Skills Therapy
- Sensory therapy
- No therapy  (8)
- Other (Please indicate)  (7) _________________________________

Q12 My child with ASD attends a:

- Mainstream kindergarten/preschool/early years setting or school  (with typically developing kids)
- Special centre/school
- Private setting (with typically developing kids)
- Homeschooling with a parent or private teacher
- No specific schooling

Q13 Please indicate your level of agreement with the following statement:

|  | Strongly disagree | Somewhat disagree | Neither agree nor disagree | Somewhat agree | Strongly agree |
| --- | --- | --- | --- | --- | --- |
| The pandemic severely affected my child's well-being |  |  |  |  |  |

Q14   How do you rate overall mood during the pandemic?

|  | Strongly negative | Negative | Neutral | Positive | Strongly positive |
| --- | --- | --- | --- | --- | --- |
| Yours |  |  |  |  |  |
| Your child`s |  |  |  |  |  |

Q15 What kind of challenges relate to your child's behaviours during the pandemic? Please select all answers that apply.

- Stereotypical behaviour and lack of interest
- Anxious behaviours (e.g. self-harm, excessive worry,  aggression, anger attacks)
- Problems in communication and social interactions
- Physical problems (unstable walking, lacing shoes)
- Mastering daily life skills (e.g. sleeping, feeding)
- Hyperactive behaviours (e.g. running around, interrupting others)
- Lack of safety awareness

**End of Block: CHILD BEHAVIORS BEFORE AND DURING PANDEMIC**

**Start of Block: SUPPORT**

Q16 Did your child receive autism therapy before March 2020?

- Yes
- No

Q16.1 Please select the option that best describes.

|  | Once a year | Once a month | Once a week | Several times | Every day |
| --- | --- | --- | --- | --- | --- |
| How often did you receive an autism therapy before March 2020? |  |  |  |  |  |

Q17 Have your child received an autism therapy after March 2020?

- Yes
- No

Q17.1 Please select the option that best describes.

|  | Once a year | Once a month | Once a week | Several times | Every day |
| --- | --- | --- | --- | --- | --- |
| How often has your child received autism therapy after March 2020? |  |  |  |  |  |

Q18 Please tell us how satisfied you were and are with the autism therapy your child receive(d) before and during the pandemic.

|  | Extremely satisfied | Satisfied | Neither satisfied nor dissatisfied | Dissatisfied | Extremely dissatisfied | Not applicable |
| --- | --- | --- | --- | --- | --- | --- |
| Before March 2020 |  |  |  |  |  |  |
| After March 2020 |  |  |  |  |  |  |

Q19  Does your child have online therapeutic support during the pandemic?

- Yes
- No

Q19.1 Please select the option that best describes.

|  | Extremely good | Somewhat good | Neither good nor bad | Somewhat bad | Extremely bad |
| --- | --- | --- | --- | --- | --- |
| How would you rate the quality of online therapy? |  |  |  |  |  |

Q19.2

|  | Definitely not | Probably not | Might or might not | Probably yes | Definitely yes |
| --- | --- | --- | --- | --- | --- |
| Would you use online support services (e.g.therapy) after the pandemic? |  |  |  |  |  |

Q20 How do you rate the importance of the types of support for your child’s well-being during a pandemic?

|  | Extremely important | Very important | Moderately important | Slightly important | Not at all important |
| --- | --- | --- | --- | --- | --- |
| Family support |  |  |  |  |  |
| Educational services |  |  |  |  |  |
| Social informative support |  |  |  |  |  |
| Regular therapy |  |  |  |  |  |
| Consultations with therapists |  |  |  |  |  |

Interview. If you would like to participate in interviews on this topic, please indicate your telephone number or email address in the box.

________________________________________________________________

**Interview protocol**

*Introduction*

As you already know, we`re conducting a study with parents of children with autism to identify the effects of the Covid-19 pandemic on your child as well as your parenting experience. The interview consists of three parts: before, during, and after quarantine. If you feel uncomfortable with some questions, you can skip them. Please do not feel obliged to answer all of them. With your permission, we will record the sessions. Are you okay with it?

*Main part*

First, let`s talk about your life experiences before the quarantine, which dates back to March 2020.

1. Where did you live before quarantine? (probe: I was a student living in Astana before March 2020)
2. How was your child's well-being at that time? What were the regular behaviors? What kind of regular activities did your child do at home? (e.g. play)
3. Was there a daily plan your child followed before the pandemic? Please describe.

Now we move on to discussing life during quarantine, which lasted for about 6 months between March and August 2020.

1. Did you live in the same place when the quarantine was announced? What kind of restrictions were there? (Probe: I had to move to another city due to the pandemic. The city council imposed strict restrictions from March to September. They then eased the restrictions. Mask wearing was formally mandatory, but not all people did follow rules)
2. Did the quarantine restrictions affect you? (e.g. social gatherings, daily activity) If yes, in what ways?

5.1. Did the quarantine restrictions affect your child? If yes, in what ways?

5.2. Could you give us examples of pandemic-caused changes in your child's physical health?

5.3  Could you also comment on any changes you did notice about your child's mental well-being?

1. Please provide us with an example of what has worked well to support your child during the pandemic. (Probe: ask for examples)
2. Did a daily plan your child follow change during the quarantine/lockdown? (e.g. such as meals and naps). What was different from the regular plan you had before?

7.1 How did you spend your time with your child during the quarantine? Are you aware of plays and activities that can support your child?

1. Were you able to access therapy or any educational content during the quarantine? What were they?
2. Have you had a chance to receive therapy online, for instance, using Zoom or Whatsapp?

If yes:

9.1 What do you think about telehealth and other online services provided as an alternative to autism therapy?

9.2 How do you explain the difference between traditional and online therapy?

1. What do you think about whether the pandemic affects your daily parenting experience? If yes, in what ways?

8.1 Could you provide examples of changes in your daily routine that may be affected by lockdown? For instance, job duties and medical checkups before or after quarantine.

1. Any positive changes during the time of lockdown?
2. Any negative changes during the time of lockdown?

Finally, we are going to talk about life after the lockdown. Although there was some difference between regions being in green, yellow, or red zones, strict lockdowns like in March 2020 were not common.

1. When have you started noticing that life is returning to normal times? (Probe: I'm working from home and still wearing masks in public places. I haven't felt any significant difference. But there were some positive signs at the end of this winter)
2. Was your child able to transition back to pre-quarantine times?
3. Any positive sustaining changes since the lockdown?

Any negative sustaining changes since the lockdown?
